# Supplementary material for: Optimized Nuclear Pellet Method for Extracting Next-Generation Sequencing Quality Genomic DNA from Fresh Leaf Tissue
Source: Methods Protoc. 2019 Jun 25;2(2):54. doi: 10.3390/mps2020054 (PMC6632156; doi:10.3390/mps2020054)
Supplement: Supplementary file 1 [file mps-02-00054-s001.pdf]

Supplementary Table 1: List of primers [18] used in qPCR for genome copy analysis.

	Gene Symbol	Forward Primer	Reverse Primer
Nuclear genome	Actin 1	GGATATGCTCTCCCCCATGC	TCCCTCACAATTTCCCGCTC
	GAPDH	GGATATGCTCTCCCCCATGC	TCCCTCACAATTTCCCGCTC
Plastid genome	Atp I	CCACAAACCATCCCAACCGA	AAAGAGCACCCGACCAGTTC
	psbA	ACATCGGATGGTTCGGTGTT	GATCGCCGCAGAAGTAGGAA
Mitochondrial genome	cob	TCCTAATGTTTTGGGGCATC	AGAATGGCATGGATCGGTAG
	cox II	GCCAGAAACGGAGAGTTGAG	TCGTATATCGCTCCACCACA

Supplementary Table 2: Times required for CTAB-based, QIAGEN DNeasy Plant Mini Kit, nuclear pellet and optimized nuclear pellet methods of extracting genomic DNA. The time required was calculated with 4 samples for each extraction method.

Methods	Extraction		Purification		Total (min)
	Incubation	Centrifuge	Incubation	Centrifuge	
CTAB-based	80	35	65	10	190
QIAGEN DNeasy Plant Mini Kit	15	7	5	3	30
Nuclear pellet	185	50	-	-	235
Optimized nuclear pellet	185	50	70	55	360
